# Leveraging Machine Learning to Predict and Assess Disparities in Severe Maternal Morbidity in Maryland

**DOI:** 10.3390/healthcare13030284

**Published:** 2025-01-31

**Authors:** Qingfeng Li, Y. Natalia Alfonso, Carrie Wolfson, Khyzer B. Aziz, Andreea A. Creanga

**Affiliations:** 1 Department of International Health, Johns Hopkins Bloomberg School of Public Health, Baltimore, MD 21205, USAcwolfso2@jhu.edu (C.W.);; 2 Johns Hopkins Children’s Center, Johns Hopkins School of Medicine, Baltimore, MD 21205, USA; 3 Department of Gynecology and Obstetrics, Johns Hopkins School of Medicine, Baltimore, MD 21205, USA

**Keywords:** severe maternal morbidity, relative risks, machine learning

## Abstract

Background: Severe maternal morbidity (SMM) is increasing in the United States. The main objective of this study is to test the use of machine learning (ML) techniques to develop models for predicting SMM during delivery hospitalizations in Maryland. Secondarily, we examine disparities in SMM by key sociodemographic characteristics. Methods: We used the linked State Inpatient Database (SID) and the American Hospital Association (AHA) Annual Survey data from Maryland for 2016–2019 (N = 261,226 delivery hospitalizations). We first estimated relative risks for SMM across key sociodemographic factors (e.g., race, income, insurance, and primary language). Then, we fitted LASSO and, for comparison, Logit models with 75 and 18 features. The selection of SMM features was based on clinical expert opinion, a literature review, statistical significance, and computational resource constraints. Various model performance metrics, including the area under the receiver operating characteristic curve (AUC), accuracy, precision, and recall values were computed to compare predictive performance. Results: During 2016–2019, 76 per 10,000 deliveries (1976 of 261,226) were in patients who experienced an SMM event. The Logit model with a full list of 75 features achieved an AUC of 0.71 in the validation dataset, which marginally decreased to 0.69 in the reduced model with 18 features. The LASSO algorithm with the same 18 features demonstrated slightly superior predictive performance and an AUC of 0.80. We found significant disparities in SMM among patients living in low-income areas, with public insurance, and who were non-Hispanic Black or non-English speakers. Conclusion: Our results demonstrate the feasibility of utilizing ML and administrative hospital discharge data for SMM prediction. The low recall score is a limitation across all models we compared, signifying that the algorithms struggle with identifying all SMM cases. This study identified substantial disparities in SMM across various sociodemographic factors. Addressing these disparities requires multifaceted interventions that include improving access to quality care, enhancing cultural competence among healthcare providers, and implementing policies that help mitigate social determinants of health.

## 1. Introduction

Severe maternal morbidity (SMM), defined by the Centers for Disease Control and Prevention (CDC) as “unexpected outcomes of labor and delivery that result in significant short- or long-term consequences to a woman’s health,” has been increasing in the United States [1]. SMM carries a range of adverse consequences for women’s health, encompassing elevated medical costs, prolonged hospitalization stays, and enduring health effects [2]. Additionally, SMM is often accompanied by adverse neonatal outcomes, including preterm birth, stillbirth, and mortality [3].

The CDC utilizes a list of 21 indicators and corresponding International Classification of Diseases (ICD-10-CM) codes from administrative hospital discharge data to identify delivery hospitalizations with SMM [1]. Following its validation in medical records data, the algorithm is widely used at both national and state levels for program benchmarking [4]. A recent study, drawing on 11.6 million delivery-related hospitalizations in the United States, found that the prevalence of SMM increased from 146.8 to 179.8 per 10,000 discharges during the period 2008–2021 [4]. This SMM burden translates to an annual incidence of over 50,000 SMM cases in the United States. Disparities across racial and ethnic groups are also consistently demonstrated in both national and state data [5].

The progression of pregnancy complications to severe illness and death is largely predictable and preventable [6,7]. Studies suggest that between one-third and two-fifths of maternal adverse events can be averted [8,9,10]. Machine learning (ML) has emerged as a powerful technique, showcasing promising prediction performance in various medical fields. Compared to traditional statistical approaches, ML algorithms exhibit superiority in handling large data with numerous features and achieving high predictive accuracy. ML algorithms have only recently found applications in maternal health [11,12].

The main objective of this study is to explore the feasibility and efficacy of ML techniques to accurately predict SMM. Our research question is whether the application of ML techniques to hospital discharge records can predict delivery hospitalizations with SMM. Several modeling approaches were explored, and the results from the best-performing model are presented in this report. The secondary objective of this study is to examine disparities in SMM by key sociodemographic characteristics based on the ML approach. Such an exploration holds significant implications. Successful early detection provides opportunities for timely and appropriate intervention, which may prevent the progression of SMM or mitigate its onset and consequences. This underscores the importance of identifying at-risk individuals at the earliest possible stage to improve maternal health and reduce the burden of SMM.

In this report, we first describe the dataset utilized in our study and outline our methodologies, then present and discuss our results, and conclude with an acknowledgment of the limitations of the current research; our conclusion highlights the implications of this study and offers suggestions for future research.

## 2. Materials and Methods

We used linked data from the Agency for Healthcare Research and Quality (AHRQ)’s State Inpatient Database (SID) and the American Hospital Association (AHA) Annual Survey data from Maryland for 2016–2019. The Maryland SID includes the universe of inpatient discharge records from community hospitals in Maryland. The SID data from Maryland are superior to corresponding databases in most other states in that they include information about intensive care unit admissions, obesity status, and history of substance use and allow longitudinal linking of patients over time via a unique patient identifier. The elements of the AHA survey that we linked with the SID data include hospital demographics, facility and service-line offerings, beds, utilization, finance and staffing characteristics, hospital organization, and leadership. The analytic sample comprised all delivery hospitalizations in Maryland hospitals during this period.

CDC’s SMM algorithm was used to identify deliveries during which patients experienced SMM (excluding blood transfusion) [1]. Starting in October 2015, the International Classification of Diseases 10th Revision codes are used to identify 21 markers of organ-system dysfunction during delivery hospitalizations with SMM using administrative hospital discharge data. The algorithm is applied to delivery hospitalizations in patients aged 12–55 years.

We organized the potential features of SMM in our linked SID-AHA data into four broad domains: delivery hospital characteristics (from AHA), patients’ sociodemographic characteristics (from SID), general pregnancy risk factors (from SID), and comorbidities and pregnancy complications (from SID). Altogether, we identified 75 potential risk factors of SMM across these four domains, of which 64 are categorical and 11 are numerical variables.

We first examined the unadjusted relative risk of SMM across key sociodemographic factors (i.e., race–ethnicity, household income, insurance status, and primary language), aiming to provide insights into inequities in this outcome.

For formal modeling, there exists a wide array of options for modeling a binary target variable. Among these, the traditional and widely used approach is logistic regression, which provides a straightforward and interpretable framework for predicting binary targets. Building on this foundation, a natural extension is the LASSO (Least Absolute Shrinkage and Selection Operator) regression. LASSO not only estimates the model parameters but also incorporates feature selection by applying a penalty to the coefficients, effectively shrinking some of them to zero. This dual functionality of LASSO makes it particularly valuable in scenarios where the dataset contains a large number of features, as it helps to identify the most relevant features while simultaneously improving model interpretability and reducing overfitting. These methods, among others, offer robust tools for addressing binary classification problems in various research and practical applications.

In this study, we started with a traditional Logit regression model of SMM on the 75 features available in our dataset. Subsequently, we employed LASSO for SMM prediction. The Logit regression for a binary target variable Y∈{0,1} can be expressed as

(1) $$logit\left(P\left(Y=1|X\right)\right)=\beta X$$

where X denotes the features and β denotes the coefficients. Adding a penalty to the log-likelihood gives a LASSO regression as follows:

(2) $$\sum_{i=1}^{n}\left(y_{i}\log\left(p_{i}\right)+\left(1-y_{i}\right)\log\left(1-p_{i}\right)\right)-\lambda \sum_{j=1}^{p}\left|\beta_{j}\right|$$

where $y_{i}$ and $p_{i}$ denote the observed value and expected probability for the i-th observation, respectively; n denotes the number of records; and λ denotes the hyperparameter, which controls the strength of the penalty on the coefficients. A small λ leads to minimal penalty, allowing more coefficients to be non-zero, i.e., less feature selection and larger variance. A larger λ results in more coefficients being shrunk to zero, which aids in feature selection.

The LASSO approach serves for both variable selection and regularization in predictive analysis. By introducing a penalty term based on the absolute values of model coefficients, LASSO effectively shrinks some coefficients to zero [13]. The best value of λ was tuned using cross-validation to balance the bias–variance trade-off. This ability proves particularly advantageous in scenarios with a substantial number of features, preventing overfitting and enhancing interpretability [14]. The LASSO model outperformed the other modeling approaches we explored for the dataset, including support vector machines (SVMs) and random forests. A 10-fold leave 5% out cross-validation approach was employed to assess predictive performance. In each of the ten iterations, a randomly chosen 5% sample was excluded from model training, and out-of-sample predictions for those samples were compared with the true values.

Many of the 75 features exhibited high correlations with each other, leading to singular matrices and non-convergence in model fitting with both the Logit and LASSO models. To address the convergence issue and explore result sensitivity, the Logit, and LASSO models with a short list of 18 features were fitted and compared. Variable selection for the reduced 18-feature model was based on literature reviews [15,16], expert opinion, exploratory correlation analysis, statistical significance, and computational resource constraints. Due to the large number of records in the dataset, including too many features in the model, makes the model fitting highly computationally intensive, often exceeding the capacity of even a high-performance desktop computer. The 18 features judged to represent a minimum necessary list of features are maternal age, presence of comorbid conditions (including chronic medical conditions, obesity), substance use during pregnancy, delivery hospital’s teaching status, and annual delivery volumes. Some women contributed more than one delivery during the research period, which was accounted for by the woman-level clustering effect.

We computed multiple performance metrics, including true positive (TP), true negative (TN), false positive (FP), false negative (FN), AUC, accuracy, precision, recall score, and F1 score.

(3) $$Precision=\frac{TP}{TP+FP}$$

(4) $$Recall=\frac{TP}{TP+FN}$$

(5) $$F1=2*\frac{precision*recall}{precision+recall}$$

The F1 score is used to evaluate the performance of a classification model in scenarios where class imbalance may exist. It is the harmonic mean of precision and recall, providing a single score that balances both metrics. The F1 score ranges from 0 to 1, where 1 indicates perfect precision and recall and 0 indicates poor performance.

We used the ROC (receiver operating characteristics curve) and AUC (area under the ROC curve) to assess and compare the predictive performance of the Logit and LASSO models. An ROC curve plots two parameters (sensitivity; 1—specificity) of a prediction model at all classification thresholds. AUC measures the entire two-dimensional area under the complete ROC curve. Although several other measures exist, many are inappropriate for highly imbalanced datasets where one target value is much more frequent than the other. Both the Logit and LASSO models were estimated in R (version 4.4.0). The model fitting was performed on a desktop computer (Windows 11; CPU i9-12900KF; 64 G RAM). The LASSO model with 18 features took about 10 min to converge, while the Logit model took less than 1 min.

Data are from public de-identified databases. For this reason, this study was exempt from review by the Institutional Review Board at our institution.

## 3. Results

### 3.1. SMM Rates

Our analytic dataset included 261,226 delivery hospitalizations in Maryland during 2016–2019. The SMM rate was 0.76 per 10,000 deliveries, ranging between 0.55 and 1.12 per 10,000 deliveries among non-Hispanic White and non-Hispanic Black patients.

The unadjusted relative risk (RR) of SMM varied markedly across different racial and ethnic groups. Non-Hispanic Black women face a substantially higher risk of experiencing SMM compared to non-Hispanic White women (RR = 2.03). Hispanic and non-Hispanic other groups also experience elevated risks (RR = 1.18 and 1.27, respectively). We also found a linear relationship between SMM and median household income for the residence area, with women in the lowest income quartile having significantly higher risks of SMM than women in the highest quartile. Women with public insurance have the highest relative risk of SMM (RR = 1.36) compared to those with private insurance. Also, women whose primary language is not English face a marginally higher risk (RR = 1.06) compared to English speakers.

### 3.2. Logit Modeling

**Figure 1:**
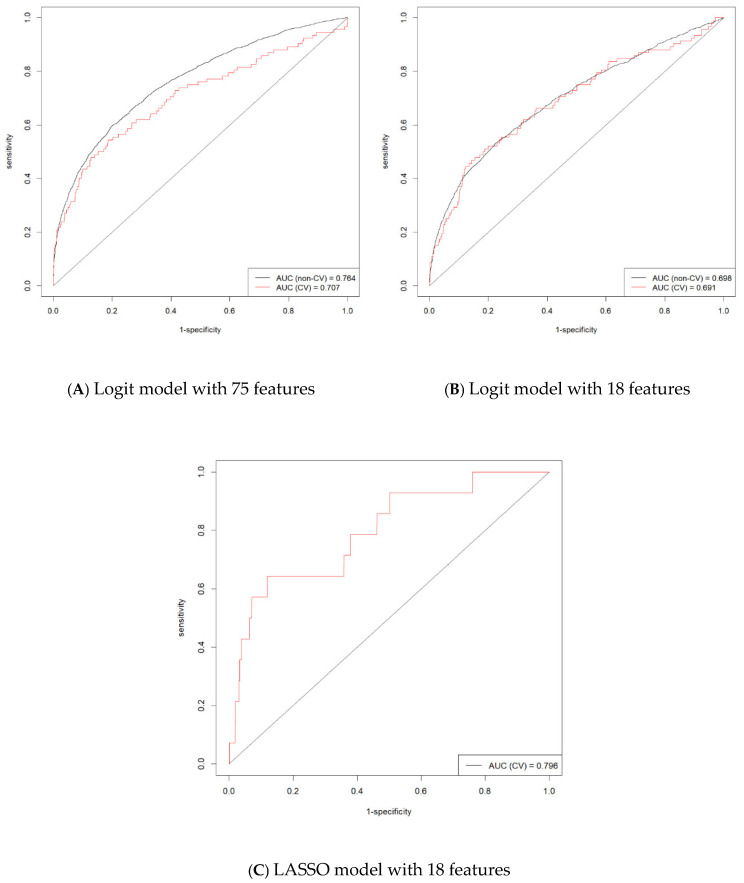
Severe maternal morbidity predicting performance for the Logit models with 75 features and the Logit and LASSO models with 18 features. Notes: Reduction from 75 to 18 features based on literature reviews, exploratory correlation analysis, statistical significance, and computational resource constraints. The 18 features include year, maternal age, primary language, insurance status, homeless status, median household income quartile for the patient zip code, level of maternal care, teaching status of the hospital, hospital contracts with payors being tied to performance on quality/safety measures, hospital has patient/family advisory, hospital encounter in the past 30 days before delivery hospitalization, obesity, multiple gestations, supervision of high-risk pregnancy, hypertensive disease, comorbidities, annual delivery volume for the hospital, and % of deliveries to minority (non-White non-Hispanic) women in the hospital.

For the 75-feature Logit model, the AUC for non-cross-validation (non-CV) was 0.764, and the AUC for the cross-validation was 0.707 (Figure 1A). The Logit model with 18 features achieved comparable predictive performance, with a non-CV AUC of 0.698 and a CV AUC of 0.691 (Figure 1B). We used the default threshold of 0.5 for the binary classification task when calculating the metrics reported in Table 1. This threshold implies that predictions with a probability of 0.5 or higher were classified as positive, while those below 0.5 were classified as negative. The choice of this default threshold reflects standard practice in binary classification tasks, providing an initial evaluation of model performance.

### 3.3. Comparison Between Predicting Performance with LASSO vs. Logit Modeling

The LASSO model with the full list of 75 features did not converge, while the model with 18 features did. Non-CV essentially represents in-sample validation, where the whole dataset is used in both model training and validation. Including the prediction dataset in training usually increases the AUC, making non-CV a reference for model performance.

Compared with the Logit models, LASSO achieved a higher CV AUC of 0.796 (Figure 1C). Table 1 presents model performance metrics for the validation dataset of 13,070 deliveries, representing a 5% random sample of the total 261,226 deliveries in the full dataset. Overall, all models exhibited good predictive performance. LASSO’s precision score of 0.64 signifies that 64% of instances predicted as positive are indeed true SMM. A precision score of this magnitude is indicative of an acceptable false positive rate. Across all performance metrics, LASSO consistently outperforms the Logit models.

Recall values remain low in all models, suggesting low sensitivity and a limited ability to identify all positive cases. This raises concerns and underscores the need for further research. The persistently low recall values are a common target value in machine learning classification tasks with highly skewed data [17,18]. The high precision and low recall values imply that the optimal application of the prediction algorithm is for initial screening rather than final confirmation.

### 3.4. Comparison with Previous Models

Our model demonstrates performance that is comparable to, or in some cases superior to, existing models documented in the literature (Table 2). However, model features included and metrics reported vary significantly across different works, making a direct and comprehensive comparison challenging.

## 4. Discussion

Our findings indicate that both the Logit and LASSO models can effectively predict SMM with acceptable accuracy, even with a relatively short list of features. The AUC values are comparable to similar applications in maternal health [28,29,30]. The LASSO model outperformed the Logit model in terms of predictive metrics, although the margin of outperformance is small. This may be attributed to the complex nature of SMM [31,32,33] and highly imbalanced data, with SMM prevalence <1%. ML models, by nature, learn much more about non-SMM cases than SMM cases. However, methodologically, this study employs an ML technique that is unconstrained by the preset assumptions of statistical distributions regarding variables and parameters. To our knowledge, this is one of few attempts to predict SMM using ML techniques [19] and the only study to employ LASSO. Hence, our results can serve as a comparison for future ML applications to predict SMM and other adverse maternal health outcomes.

**Figure 2:**
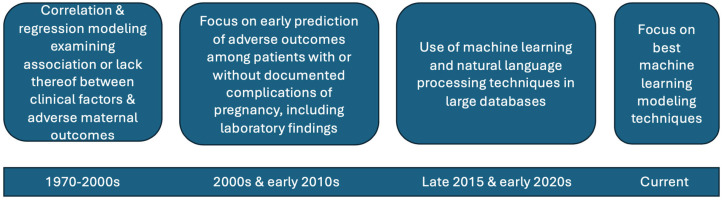
Efforts to predict maternal morbidity and severe outcomes over time.

Our scoping review of the literature shows how the prediction of maternal health outcomes varies and changes over time (Figure 2). Studies in the 1970s until the 2000s employed correlation and regression analyses to examine associations (or lack thereof) between specific clinical factors and adverse maternal outcomes [34,35,36]. The 2000s and 2010s brought an interest in early prediction of adverse outcomes in patients with or without documented pregnancy complications using clinical and laboratory tests [37,38,39]. The use of ML and NLP techniques is relatively new in maternal health, likely due to limitations in the data available and the fact that adverse outcomes are statistically rare events [24,40,41,42,43]. Currently, the focus is on identifying the best-performing ML techniques for predicting outcomes, such as SMM, and this is where our study makes a contribution [44,45].

Some studies aim to predict maternal outcomes to improve clinical care by evaluating new or improved screening guidelines or tools (e.g., the inclusion of proteinuria) [37,38,39,46,47,48,49]. Others assess the effect of potential features available through advances in technology or data access (e.g., placental imaging techniques, genetic data, novel biomarkers such as placental growth factor, platelet parameters, plasma cell-free RNA) [34,35,36,43,50,51,52,53,54,55,56,57]. And, only in the past decade or so have studies started to compare the predictive power of different statistical classification models (i.e., ML models, logistic regression, naive Bayes, adaptive models) [24,40,44,58,59,60].

Studies can also be distinguished by the timing of screening. Among those predicting preeclampsia risk in pregnant women using first-trimester data, some have used clinical and laboratory data to compare the predictive power of different classification models. These studies show that models can perform well, with AUCs as high as 0.89 (using the elastic net algorithm) or 0.86 (using random forest) [41,60]. Similar studies examining biomarkers, specifically systolic blood pressure polygenic risk scores, suggest this feature does not improve preeclampsia predictions [45]. Other studies combining multiple biomarkers (e.g., placental growth factor, clinical, and biophysical features) demonstrate that this combination improves predictions compared to PLGF alone (AUC 0.937 with logistic regression) [39]. Additional studies using first-trimester data provide more insights into biomarker quality assessment (e.g., cumulative sum and target plots to assess blood pressure or placental growth factor) [48]. Studies assessing features obtained during the second trimester also looked at blood pressure to predict the risk of hypertension or preeclampsia. These studies include some of the earliest prediction models for pregnant women’s risks and found evidence for predicting hypertension but not preeclampsia [34,35]. Other studies assess specific biomarkers, irrespective of the trimester of data collection, including platelet parameters or plasma cell-free RNA data [42,57]. More recent studies evaluating features obtained during the second or third trimesters focused on the risk of other pregnancy outcomes, postpartum hemorrhage, SMM, or fetal outcomes [24,46,49,55,61]. A recent study examining the risk of SMM assessed the predictive power of natural language processing (NLP) models using history and physical note texts (excluding additional patient information, such as demographics and diagnosis codes) and obtained an AUC of 0.76 [24].

The remaining studies assessed the risk of adverse pregnancy outcomes among women diagnosed with preeclampsia. The most recent study comparing different classification models found that among the nine models tested, elastic net, stochastic gradient boosting, extreme gradient boosting, and random forest produced the strongest discrimination between true and false outcomes, with AUCs between 0.860 and 0.973 [44]. Other studies evaluated predictions with specific biomarkers (e.g., liver function tests, creatinine concentrations from lab data, placental growth factors) [36,43,50]. Among these, the strongest performing model achieved an AUC of 0.88 using random forest [43]. Additional studies used logistic regression to evaluate the effect of routine biomarkers and clinical characteristics data on prediction performance, and these models achieved AUCs ranging between 0.71 (with external validation) and 0.88 or 0.91 with and without cross-validation, respectively [37,38,62].

Our study also reveals significant inequities in SMM across key sociodemographic factors. Women from low-income households, non-Hispanic Black women, those with public insurance, and non-English speakers are particularly vulnerable. Addressing these disparities requires multifaceted interventions that include improving access to quality care, enhancing cultural competence among healthcare providers, and implementing policies that mitigate the social determinants of health. By focusing on these areas, we can work towards a more equitable maternal healthcare system that ensures all women have the opportunity for safe and healthy pregnancies.

This study has several limitations. First, the ML algorithms are trained and validated using only data from Maryland. They may need further fine tuning using more diverse datasets to enhance generalization performance. Such external validation on an entirely separate dataset from a different source or population could provide further insights. However, such datasets were not available to us. Secondly, we explored several other ML models that we identified through our literature search, including decision trees, random forests, K-Nearest Neighbors (KNNs), and support vector machines (SVMs). Although these models have been extensively applied in classification tasks, they either did not converge or performed poorly in classifying SMM in our data. This is likely due to the highly imbalanced distribution of the SMM outcomes in our data. Thirdly, there are more advanced ML algorithms, such as deep neural networks, which offer greater modeling flexibility and predictive power. However, these sophisticated methods come with their own set of limitations, including increased complexity, higher computational costs, and challenges in implementation and interpretation. As a result, they fall outside the scope of the present study, which focuses on more accessible and interpretable approaches. Those advanced methods can be explored in future research.

## 5. Conclusions

Our study highlights the potential of ML algorithms in predicting SMM in large databases. More research is warranted to validate and refine these algorithms and achieve more accurate SMM detection. Potential directions for future research include leveraging advanced methods, such as the Synthetic Minority Oversampling Technique, to address the highly imbalanced nature of the data, or employing sophisticated deep neural networks to enhance predictive performance. Our study also confirms substantial equity issues in maternal health across various sociodemographic aspects in Maryland. By focusing on these areas, we can work towards a more equitable maternal healthcare system that ensures all women have the opportunity for safe and healthy pregnancies.

## Figures and Tables

**Table 1:** Performance of the Logit and LASSO models in predicting severe maternal morbidity using hospital discharge data.

Algorithm	TP	FP	TN	FN	AUC	Accuracy	Precision	Recall
Logit-75	50	42	10559	2419	0.71	0.81	0.54	0.02
Logit-18	44	48	10979	1999	0.69	0.84	0.48	0.02
LASSO-18	45	25	11454	1546	0.80	0.88	0.64	0.03

Notes: TP = true positive; FP = false positive; TN = true negative; FN = false negative; AUC = area under the receiver operating characteristic curve. Accuracy refers to the proportion of correct predictions out of the total number of predictions. Precision refers to the proportion of correctly predicted positive cases out of all cases predicted as positive. Recall refers to the proportion of actual positive cases that were correctly predicted by the model.

**Table 2:** A Summary of the predictive performance of machine learning models for SMM.

Article	Algorithm	Precision	Recall	AUC or F1
Gao 2019 [19]	Regularized Logit	0.22 to 0.35	Sensitivity: 0.614 to 0.765	AUC: 0.790 to 0.937
Rodríguez 2016 [20]	Logit	NA	NA	AUC 0.66
Xu 2023 [21]	EBM; Logit	NA	0.59	AUC EBM 0.70 AUC Logit 0.69
Lengerich 2024 [22]	GAM	0.0152	0.369	AUC: 0.67
Rodríguez 2020 [23]	Logit; SVM	Logit: 0.518; SVM: 0.279	Logit: 0.977: SVM: 1	F1: Logit 0.677 vs SVM: 0.436
Clapp 2022a [24]	LASSO with BoW	NA	NA	AUC for SMM: 0.67–0.72; AUC for NT SMM: 0.72–0.76
Clapp 2022b [25]	NLP with BoW; LASSO with BoW	0.194 (SMM); 0.084 (NT SMM)	0.287 (SMM); 0.298 (NT SMM)	AUC for SMM: 0.76; AUC for NT SMM: 0.75
Clapp 2021 [26]	LASSO; Elastic Net, Ridge	0.075	0.11	AUC 0.611
Leonard 2022 [27]	Logit	NA	NA	AUC: 0.68–0.76

Notes: BoW, bag of words; EBM, explainable boosting machine; GAM, Generalized Additive Model; NLP, natural language processing; NPV, negative predictive value; NT SMM, non-transfusion SMM; SPR, screen positive rate.

## Data Availability

The original data presented in the study are openly available through AHRQ’s HCUP project.
